# Rapid detection of Mucorales based on recombinase polymerase amplification and real-time PCR

**DOI:** 10.3389/fmicb.2023.1273073

**Published:** 2023-10-20

**Authors:** Rui Xu, Dingchen Li, Jingya Zhao, Hanying Zhong, Hong Chen, Yajing Jia, Fangyan Chen, Li Han

**Affiliations:** ^1^Department for Disinfection and Infection Control, Chinese PLA Center for Disease Control and Prevention, Beijing, China; ^2^School of Public Health, China Medical University, Shenyang, Liaoning, China; ^3^College of Public Health, Zhengzhou University, Zhengzhou, Henan, China

**Keywords:** mucormycosis, order Mucorales, RPA, real-time PCR, COVID-19, diagnosis

## Abstract

Mucormycosis, an invasive fungal disease with severe consequences, poses a significant threat to immunocompromised individuals. However, the timely and accurate identification of Mucorales infection continues to present difficulties. In this study, novel detection techniques utilizing recombinase polymerase amplification (RPA) and quantitative real-time polymerase chain reaction (qPCR) were developed, specifically targeting the mitochondrial *rnl* gene, in order to address this challenge. The specificity of the RPA and qPCR assay was assessed by adding genomic DNAs extracted from 14 non-targeted strains, as well as human and mouse blood. No false-positive results were observed. Additionally, genomic DNAs from 13 species in five genera of order Mucorales were tested and yielded positive results in both methods. To further evaluate the sensitivity of the assays, DNAs from *Rhizopus oryzae, Mucor racemosus, Absidia glauca, Rhizomucor miehei,* and *Cunninghamella bertholletiae* were utilized, with concentrations ranging from 1 ng/μL to 1 fg/μL. The limit of detection (LoD) for the RPA assay was determined to be 1 pg., with the exception of *Rhizomucor miehei* which had a LoD of 1 ng. The LoD for the qPCR assay varied between 10 fg and 1 pg., depending on the specific species being tested. Sensitivity analysis conducted on simulated clinical samples revealed that the LoD for RPA and qPCR assays were capable of detecting DNA extracted from 10^3^ and 10^1^ colony forming units (CFU) conidia in 200 μL of blood and serum, respectively. Consequently, the real-time RPA and qPCR assays developed in this study exhibited favorable sensitivity and specificity for the diagnosis of mucormycosis.

## Introduction

Mucormycosis is a perilous and extensively damaging invasive fungal disease caused by filamentous fungi belonging to the order Mucorales, primarily affecting individuals with compromised immune systems (Millon et al., 2019; Guegan et al., 2020). Additional predisposing risk factors include *diabetes mellitus*, recipients of hematopoietic stem cell transplants, severe traumatic injuries, burns, and hematological malignancies. The majority of mucormycosis cases arise from the inhalation of fungal sporangiospores or direct inoculation through wounds, subsequently leading to the growth of angioinvasive hyphae (Skiada et al., 2018). The order Mucorales encompasses a vast array of species, totaling more than 250 across 55 genera (Badali et al., 2021). Among these, the primary pathogens responsible for human mucormycosis are *Rhizopus* spp., *Mucor* spp., *Lichtheimia* spp. (formerly known as *Absidia*). Additionally, *Rhizomucor* spp., *Cunninghamella* spp., *Apophysomyces* spp., and *Saksenae* spp. have also been identified as causative agents (Badali et al., 2021). It is worth noting that the etiology of mucormycosis exhibits regional variation, with *Rhizopus* spp., *Mucor* spp., and *Lichtheimia* spp. being the most prevalent in European patients. In India, *Rhizopus* species are the predominant pathogens, including *Apophysomyces elegans, Apophysomyces variabilis* and *Rhizopus homothallicus*, which have also emerged as significant contributors (Skiada et al., 2018).

The incidence of mucormycosis has experienced a substantial increase in recent decades due to the expanding number of susceptible populations (Ambrosioni et al., 2010; Saegeman et al., 2010; Guinea et al., 2017). Based on calculations from the Leading International Fungal Education portal, the annual prevalence of mucormycosis worldwide, excluding India, is estimated to be approximately 10,000 cases (Chander et al., 2018). However, in India, the prevalence is approximately 70 times higher than the global data, with an incidence rate of approximately 0.14 per 1,000 population. The Corona Virus Disease 2019 (COVID-19) pandemic has resulted in a notable increase in the incidence of mucormycosis. Atul Patel’s research revealed a 2.1-fold rise in mucormycosis cases in India between September and December 2020 compared to the corresponding period in 2019 (Patel et al., 2021), with COVID-19 being identified as the primary contributing factor. A comprehensive analysis of 80 cases of COVID-19-associated mucormycosis (CAM) across 18 countries, demonstrated a mortality rate of 49%, with surviving patients experiencing significant long-term impairments, such as vision loss in 46% (Hoenigl et al., 2022) of cases. Furthermore, a recent study conducted in France reported a remarkably high mortality rate of 88% within a 12-week timeframe, underscoring the potential lethality of mucormycosis as a complication of COVID-19 (Danion et al., 2022).

The most prevalent clinical presentations of mucormycosis include rhino-orbito-cerebral, pulmonary, cutaneous, gastrointestinal, and disseminated forms. Typically, symptoms manifest abruptly, leading to rapid disease progression and a high mortality rate within a short period of time (Steinbrink and Miceli, 2021). Consequently, early detection and subsequent administration of antifungal therapy are imperative for a favorable prognosis. Presently, the diagnosis of mucormycosis primarily relies on mycological culture and histopathological examination. Under direct microscopy, the clinical specimens of Mucorales are characterized with the hyphae of a variable width (ranging from 6 to 25 μM), non-septate or pauci-septate and an irregular, ribbon-like branching, and wide-angle (90°) bifurcations (Skiada et al., 2018). The potential impact of friable hyphae damage during tissue manipulation on the low sensitivity and high rate of false negative outcomes has been identified as a contributing factor (Bougioukas et al., 2022). Additionally, the suboptimal sensitivity of culture, with only approximately 50% of specimens yielding positive results even in cases of positive microscopy, may lead to delays or missed diagnoses of infections (Skiada et al., 2020). Furthermore, clinical and radiological features are unable to differentiate between various causes of invasive fungal diseases (IFD), including Aspergillosis, though recent studies have indicated that the presence of a reverse halo sign may suggest a Mucorales infection (Baldin et al., 2018). The identification of mucormycosis is not aided by the detection of galactomannan or beta-glucan (Guegan et al., 2020). Given that a delay of 6 days in diagnosis has led to a significant increase in 30-day mortality rates from 35 to 66%, it is crucial to establish prompt and accurate methods for early detection (Werthman-Ehrenreich, 2021).

Molecular methods commonly used in research include conventional polymerase chain reaction (PCR), sequencing of the amplicons, restriction fragment length polymorphism (RFLP), quantitative real-time PCR (qPCR), and high-resolution melting (HRM) analysis of PCR products. The primers used in these methods target specific regions such as the internal transcribed spacer (ITS1 and ITS2) region (Bernal-Martínez et al., 2013), the 18S rRNA gene (Scherer et al., 2018), the 28S rRNA gene (Springer et al., 2016), the high-affinity iron permease (FTR1) gene (Nyilasi et al., 2008), the mitochondrial gene rnl (Caramalho et al., 2019), the cytochrome b gene (Hata et al., 2008), and the Mucorales-specific spore coating encoding protein (cotH) gene (Baldin et al., 2018). Numerous studies have utilized formalin-fixed, paraffin-embedded (FFPE) or fresh tissue samples, serum, blood and BALF samples (Millon et al., 2013; Bellanger et al., 2018; Scherer et al., 2018) to investigate this matter, with varying levels of sensitivity (62 to 100%) and specificity (89 to 100%). A detailed overview of molecular tools published Supplementary Table S1 provides further insight into this topic (Bialek et al., 2005; Hsiao et al., 2005; Nagao et al., 2005; Machouart et al., 2006; Kasai et al., 2008; Massire et al., 2013; Lengerova et al., 2014; Alanio et al., 2015; Salehi et al., 2016; Springer et al., 2016; Yang et al., 2016; Ino et al., 2017; Ashraf et al., 2022; Bigot et al., 2022).

Presently, there exist a minimum of three commercially available qPCR methods that have been documented for the purpose of detecting Mucorales. One such method, the MucorGenius kit, has been subjected to testing and has demonstrated commendable levels of sensitivity (ranging from 75 to 90%) and specificity (100%) when applied to clinical samples (Dannaoui, 2022). Additionally, Dolatabadi et al. (2014) have devised a rolling circle amplification (RCA) technique that relies on the ITS region for the molecular identification of six distinct species of Mucorales. This technique exhibits a rapid turnaround time of 2 h and is visualized through the utilization of agarose gel electrophoresis (Dolatabadi et al., 2014). When compared to conventional detection methods, these techniques have significantly decreased the time required and are likely to enhance outcomes by facilitating timely intervention with appropriate treatment. However, the clinical application of these techniques is restricted due to their reliance on a thermal cycler. Additionally, certain studies lack comprehensive data on the limit of detection (LoD), sensitivity, specificity, and cross reactivity. The limited number of clinically validated cases necessitates further investigation for a comprehensive evaluation of these established methods.

Recombinase polymerase amplification (RPA) is an isothermal nucleic acid amplification technique that functions within a consistent temperature range of 25–40°C, completing the amplification of the target sequence in a time frame of 10–20 min (Ghosh et al., 2018). The initiation of amplification occurs through the pairing of primers with homologous sequences on the template by a recombinase. Subsequently, the reaction is stabilized by the single strand binding protein, which binds to the displaced DNA strand and prevents primer dissociation. Finally, the extension step is carried out by the strand displacing polymerase (Wang et al., 2021). The primary methods utilized for detecting RPA products include real-time fluorescence, lateral flow strips (LFS), and agarose gel electrophoresis. The majority of necessary reagents are readily available in a dehydrated pellet form. Recent literature has demonstrated the successful application of RPA-based techniques in the identification of various fungal species, such as *Candida albicans* (Wang et al., 2021), *Candida tropicalis* (Wang et al., 2022), *Candida glabrata* (Wang et al., 2022)*, Cryptococcus neoformans/C. gattii* (Ma et al., 2019), *Aspergillus fumigatus* (Li et al., 2022), and *Fusarium graminearum* (Lei et al., 2022; Liang et al., 2022; Wang et al., 2022), thereby fulfilling the demand for rapid, specific, and highly sensitive detection within a timeframe of 20–30 min (Supplementary Table S2). However, to date, there have been no published studies investigating the use of RPA for identifying fungi belonging to the order Mucorales. In this study, two detection approaches were devised for the identification of order Mucorales using probe-based real-time RPA and qPCR in order to establish a technical reference for the prompt clinical detection of mucormycosis.

## Results

### Specificity validation

**Figure 1:**
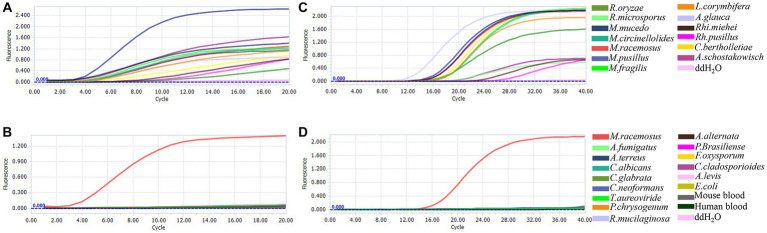
Analytical specificity of Mucorales in real-time RPA and real-time PCR assay. Genomic DNAs from 13 species in five genera of order Mucorales and 16 non-Mucorales stains, were used as template for real-time RPA reaction mixtures **(A,B)** and real-time PCR reaction mixtures **(C,D)**.

The specificity of the RPA and qPCR assay, which targeted the mitochondrial *rnl* gene, was evaluated using 13 species from various genera within the order Mucorales, as well as 14 non-targeted strains (Table 1) and samples of human and mouse blood. All 13 Mucorales strains exhibited positive signals (Figures 1A,C), while the 14 non-Mucorales strains and the human and mouse blood samples did not yield amplification in either the RPA or qPCR assays (Figures 1B,D). These findings suggest that there were no instances of false-positive amplifications with non-Mucorales species in either assay, indicating that the primer and probe combination, Mt. RPA F/R/P and Mt. qPCR F/R/P, demonstrated good interspecies specificity.

**Table 1:** Strains used in this study.

Genus	Latin name	Strain	RPA assay	qPCR assay
*Rhizopus* spp.	*Rhizopus oryzae*	Isolated strain	+	+
	*Rhizopus microsporus*	Isolated strain	+	+
*Mucor* spp.	*Mucor mucedo*	BNCC336219	+	+
	*Mucor circinelloides*	BNCC147484	+	+
	*Mucor racemosus*	BNCC336225	+	+
	*Mucor pusillus*	CICC41069	+	+
	*Mucor fragilis*	Isolated strain	+	+
*Lichtheimia* sp.	*Lichtheimia corymbifera*	Isolated strain	+	+
*Absidia* sp.	*Absidia glauca*	Isolated strain	+	+
*Rhizomucor* spp.	*Rhizomucor miehei*	BNCC337563	+	+
	*Rhizomucor pusillus*	CICC41598	+	+
*Cunninghamella* sp.	*Cunninghamella bertholletiae*	Isolated strain	+	+
*Actinomucor* sp.	*Actinomucor elegans*	BNCC336123	+	+
Non-targeted	*Aspergillus fumigatus*	Isolated strain	−	−
	*Aspergillus terreus*	Isolated strain	−	−
	*Trichoderma aureoviride*	Isolated strain	−	−
	*Penicillium chrysogenum*	Isolated strain	−	−
	*Candida albicans*	Isolated strain	−	−
	*Candida glabrata*	Isolated strain	−	−
	*Rhodotorula mucilaginosa*	Isolated strain	−	−
	*Cryptococcus neoformans*	Isolated strain	−	−
	*Alternaria alternata*	Isolated strain	−	−
	*Paraconiothyrium brasiliense*	Isolated strain	−	−
	*Fusarium oxysporum*	Isolated strain	−	−
	*Cladosporium cladosporioides*	Isolated strain	−	−
	*Acrophialophora levis*	Isolated strain	−	−
	*Escherichia coli*	Isolated strain	−	−

### Sensitivity assay

**Figure 2:**
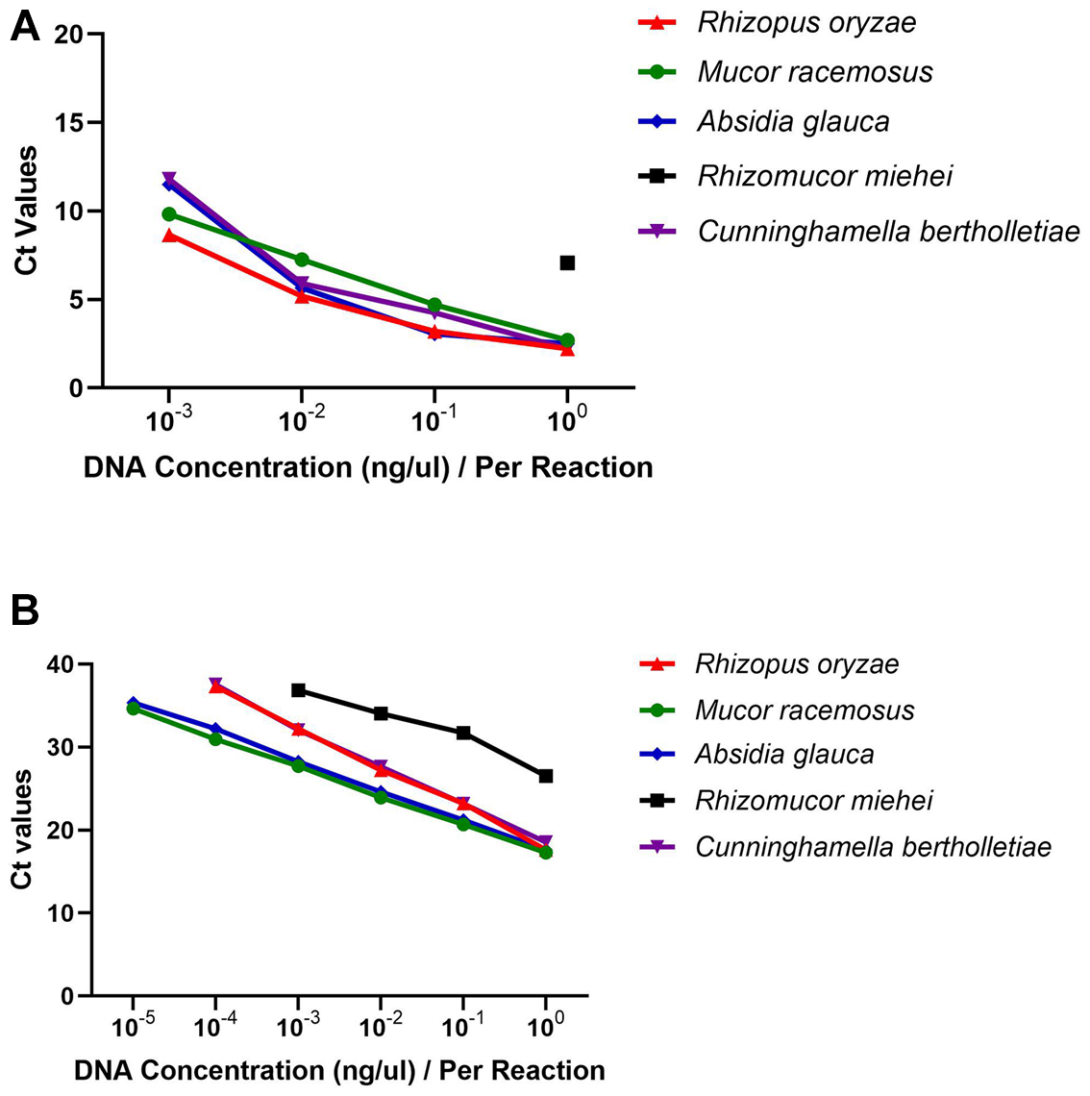
Linearity and dynamic range. **(A)** Real-time RPA: 10^−3^-10^0^ ng/μL genomic DNAs were introduced. **(B)** Real-time PCR: 10^−5^-10^0^ ng/μL genomic DNAs were introduced.

The sensitivity of the real-time RPA and qPCR assay was assessed by conducting tests on 10-fold serial dilutions of genomic DNAs from *R. oryzae*, *M. racemosus*, *A. glauca*, *Rh. miehei* and *C. bertholletiae*. The real-time RPA assay demonstrated a detection limit of 1 pg./reaction for the mitochondrial *rnl* gene, with the exception of *Rh. miehei* which had a detection limit of 1 ng/reaction (Figure 2A). The detection limits of qPCR assay were 10 fg/reaction for *M. racemosus* and *A. glauca*, 100 fg/reaction for *R. oryzae* and *C. bertholletiae*, and 1 pg./reaction for *Rh. miehei* (Figure 2B). The presence of *A. fumigatus* or *C. albicans* genomic DNA did not impact the detection limits of either assay in any of the aforementioned strains (Supplementary Tables S3A,B).

### Detection of simulated clinical samples

The simulated clinical samples comprised of a ten-fold dilution series ranging from 1 × 10^6^ to 1 × 10^1^ conidia of *R. oryzae*, *M. racemosus, A. glauca, Rh. miehei,* and *C. bertholletiae*, mixed with 200 μL of blood or serum. In terms of the simulated serum samples, the limits of detection (LoDs) of RPA were determined to be 10^3^ CFU conidia/reaction for *M. racemosus* and *A. glauca*, 10^4^ CFU for *C. bertholletiae*, 10^5^ CFU for *R. oryzae*, and 10^6^ CFU for *Rh. miehei* (Table 2A). Conversely, the LoDs of qPCR were 10^1^ CFU conidia/reaction for *R. oryzae, M. racemosus, A. glauca,* and *C. bertholletiae*, and 10^3^ CFU for *Rh. miehei* (Table 2B). Regarding simulated blood samples, the LoDs of RPA were determined to be 10^4^ CFU conidia/reaction for *M. racemosus*, 10^5^ CFU for *R. oryzae, A. glauca* and *C. bertholletiae*, and 10^6^ CFU in *Rh. miehei* (Table 2A), while the LoDs of qPCR were found to be 10^2^ CFU conidia/reaction for *R. oryzae, M. racemosus, A. glauca,* and *C. bertholletiae*, and 10^3^ CFU for *Rh. miehei* (Table 2B).

**Table 2:** LoDs (CFU) of simulated clinical samples tested by (A) real-time RPA and (B) real-time PCR^①^.

(A) Species	Saline		Serum		Blood	
	LoD	Ct Value	LoD	Ct Value	LoD	Ct Value
*R. oryzae*	10^3^	15.37 ± 2.14	10^5^	13.45 ± 1.16	10^5^	17.19 ± 3.87
*M. racemosus*	10^3^	13.27 ± 3.86	10^3^	14.79 ± 3.93	10^4^	13.89 ± 4.06
*A. glauca*	10^3^	15.69 ± 2.62	10^3^	^②^17.2	10^5^	13.20 ± 1.51
*Rh. miehei*	10^6^	^②^7.24	10^6^	^②^12.87	10^6^	11.26 ± 5.69
*C. bertholletiae*	10^4^	11.76 ± 0.79	10^4^	^②^12.98	10^5^	12.71 ± 4.72

(B) Species	Saline		Serum		Blood	
	LoD	Ct Value	LoD	Ct Value	LoD	Ct Value
*R. oryzae*	10^1^	35.91 ± 0.54	10^1^	35.66 ± 1.77	10^2^	32.86 ± 1.09
*M. racemosus*	10^1^	35.35 ± 0.63	10^1^	32.07 ± 0.82	10^2^	33.18 ± 0.68
*A. glauca*	10^1^	35.84 ± 0.79	10^1^	34.64 ± 0.75	10^2^	34.22 ± 1.15
*Rh. miehei*	10^3^	36.64 ± 0.64	10^3^	33.27 ± 2.24	10^3^	32.23 ± 3.36
*C. bertholletiae*	10^1^	36.14 ± 0.52	10^1^	36.95 ± 0.74	10^2^	32.71 ± 2.31

① Aliquots of 200 μl samples were inoculated with a 10-fold dilution series of 1 × 10^6^–1 × 10^1^ CFU conidia of *R. oryzae*, *M. racemosus*, *A. glauca*, *Rh. miehei*, and *C. bertholletiae*. Each assay was repeated in 3 different batch of samples. ② There was only one positive result among three assays.

The analysis revealed that the RPA assay exhibited lower sensitivity compared to the qPCR assay in terms of the LoDs in simulated clinical samples (Supplementary Table S4). Overall, both the RPA and qPCR assays demonstrated lower LoDs for these five species in simulated serum samples as opposed to blood samples.

## Discussion

The conventional diagnostic techniques for mucormycosis, such as pathology and culture, are predominantly invasive and may not be appropriate for specific patient populations due to their reduced sensitivity. To address this limitation, molecular assays have been proposed as supplementary tools to conventional diagnostic procedures for the detection and identification of mucormycosis (Skiada et al., 2018). In this regard, Springer et al. have developed a probe-based Mucorales-specific real-time PCR assay (Muc18S), which successfully detected Mucorales DNA in all patients with probable or proven invasive mucormycosis, achieving a detection rate of 100%. The utilization of serum samples for detection allowed for an earlier diagnosis, approximately 21 days prior to the use of tissue samples (Springer et al., 2016). Millon et al. (2022) reported a sensitivity and specificity of 85.2 and 89.8%, respectively, for serum detection. Additionally, the first positive result in serum was observed 4 days before the first mycological or histological positive specimen, and 1 day prior to the initial imaging procedure (Millon et al., 2022).

Caramalho et al. (2019) conducted a study demonstrating the potential of *rnl*, a representative gene of Mitochondrial (mtDNA), as a promising marker for diagnostic applications. This is due to the protective nature of mitochondrial DNA against degradation, its stability across multiple generations, and its higher copy numbers compared to nuclear DNA (Caramalho et al., 2019). While nuclear ribosomal genes including ITS, 18S and 28S, appear to be less suitable as pan-Mucorales markers. In particular, The ITS region shows intraspecific variability within 3.24% of the Mucoromycotina (Thiyagaraja et al., 2022). Moreover, heterogeneity in the sequences of ITS 1 and ITS 2 has been observed in *R. microspores* (Baldin et al., 2018).

The objective of our research was to develop a real-time RPA and qPCR assay utilizing probes, with the aim of rapidly detecting Mucorales by targeting the mitochondrial *rnl* gene. The results of this assay were obtained within 20 min and 40 min, respectively. The selection of *R. oryzae, M. racemosus, A. glauca, Rh. miehei* and *C. bertholletiae* for this study was based on their clinical significance. To determine the specificity of the assay, we utilized genomic DNA from 13 species within 5 genera of Mucorales, 14 non-Mucorales strains, as well as human and mouse blood samples. The real-time RPA and qPCR assays demonstrated high specificity, as no cross-reactivity was observed with other organisms. Our findings indicate that the real-time RPA assay has a detection limit of 1 pg. genomic DNA/reaction or 10^3^ CFU conidia/reaction in simulated clinical specimens. Similarly, the LoD of qPCR assay was down to 10 fg genomic DNA/reaction or 10 CFU conidia/reaction in simulated clinical specimens. The sensitivity of RPA was found to be lower than that of qPCR; however, the RPA reaction time was half as long. Importantly, both methods exhibited resistance to interference from other fungal genomic DNA (*A. fumigatus* and *C. albicans*), and their sensitivity remained unaffected.

As evidenced by the data presented in Table 2 and Supplementary Table S4, the LoDs for the simulated clinical samples was found to be lower than that of the control groups (saline groups), suggesting the influence of serum and blood. Furthermore, when comparing the sensitivity of simulated serum samples to that of simulated blood samples, it was observed that the latter exhibited a less effective sensitivity, potentially attributable to the complex composition of blood and the loss incurred during DNA extraction. Notably, in both genomic DNAs and simulated clinical specimens, the analytical sensitivity of *Rh. miehei* was significantly lower compared to the other four species.

In comparison to traditional culture methods, molecular-based assays have demonstrated the ability to provide earlier diagnosis, making them a valuable adjunct to conventional diagnostic procedures (Skiada et al., 2018). Springer et al. (2016) have described two independent probe-based real-time PCR tests for the detection of 12 Mucorales strains, with the LoDs ranging from 3 to 64 fg. Millon et al. (2013) have developed three qPCR assays for the detection of *Mucor/Rhizopus, Lichtheimia, and Rhizomucor*, with LoDs ranging from 3.7 to 15 fg/10 μL. Additionally, Dolatabadi et al. (2014) have developed a rolling circle amplification (RCA) method that has shown positive results for six of the most virulent species, with an LoD as low as 3.2 × 10^5^ copies of amplicons. In comparison to other molecular-based assays utilized for the identification of Mucorales, the qPCR assay developed in this study demonstrated an LoD as low as 10 fg, while the RPA assay exhibited an LoD as low as 1 pg. Both the RPA and qPCR methods devised in this research possess a brief turnaround time, with approximately 20 and 40 min required, respectively. In contrast, other molecular-based assays necessitated 2–24 h to obtain results (Supplementary Table S1).

The primary drawback of these two assays is their inability to identify the specific genus of clinically relevant Mucorales strains. Accurate determination of Mucorales genera is crucial for guiding comprehensive diagnosis and selecting appropriate antifungal medication, which directly impacts clinical outcomes. While amphotericin B, posaconazole and isavuconazole antifungal therapies are generally effective against Mucorales strains *in vitro*, certain genera within this order have been linked to suboptimal prognosis in terms of clinical response, potentially due to reduced susceptibility (Badali et al., 2021). For instance, studies have demonstrated that the minimal inhibitory concentration of amphotericin B against *Cunninghamella* can be as high as 40% (Almyroudis et al., 2007), resulting in a higher mortality rate compared to diseases caused by other genera (71 versus 44%) (Cornely et al., 2019; Jeong et al., 2019). Arendrup et al. (2015) discovered that triazole antifungals are less effective against *M. circinelloides* compared to other clinically relevant Mucorales species, particularly isavuconazole.

Additionally, when compared to the qPCR assay developed in this study and other molecular detection methods, the real-time RPA assay is relatively more expensive. Despite being conducted in a category II biosafety cabinet, the risk of cross contamination between samples remains significant due to the presence of numerous amplicon (Ghosh et al., 2018). Therefore, it is imperative to implement rigorous quality control measures and contamination precautions. Through further optimization, specifically enhancing analytical sensitivity and conducting comprehensive evaluations of specificity, RPA has the potential to emerge as a promising alternative to qPCR or other isothermal methods for the rapid detection of pathogenic fungi. The absence of reliance on rapid temperature ramping, as observed in PCR, renders RPA more suitable for integration into microfluidic lab-on-chip devices and amenable to field use. Consequently, RPA exhibits promising potential for rapid detection (Wang et al., 2021).

The utilization of our real-time RPA and qPCR assay in clinical samples has yet to be established, and this will be a focal point of our future research endeavors. Recent investigations have documented instances of combined aspergillosis and mucormycosis cases (Loubet et al., 2022), particularly as secondary complications in critically ill COVID-19 patients (Johnson et al., 2021; Paul et al., 2022). Consequently, the screening for specific infections or co-infections, such as aspergillosis, presents an intriguing challenge (Muthu et al., 2022; Ramani et al., 2022).

In this study, our real-time RPA and qPCR system serves as the experimental basis for the swift identification of order Mucorales in clinical specimens, thereby enhancing the diagnosis and treatment of Mucorales infection and exhibiting promising potential for practical implementation.

## Materials and methods

### Mucorales strains and growth conditions

The Mucorales strains utilized in this research were obtained from the BeNa Culture Collection, China Center of Industrial Culture Collection, and the laboratory of the Department of Disinfection and Infection Control, Chinese PLA Center for Disease Control and Prevention (as indicated in Table 1). The fungi were cultivated on potato dextrose agar at a temperature of 28°C and allowed to incubated for 2–5 days.

Sporangiospores were harvested by washing the cultures with phosphate-buffered saline (PBS) and then separated from hyphal components using a 40 μm nylon cell strainer. Following centrifugation at 3,700 × g for 5 min, the sporangiospores were washed and resuspended in PBS for subsequent specificity testing. Additionally, 5 × 10^6^ CFU sporangiospores of *Rhizopus oryzae*, *Mucor racemosus*, *Absidia glauca*, *Rhizomucor miehei*, and *Cunninghamella bertholletiae* (hereinafter abbreviated as *R. oryzae*, *M. racemosus*, *A. glauca*, *Rh. miehei*, and *C. bertholletiae*, respectively) were inoculated into 150 mL of potato dextrose medium and incubated at 28°C with agitation. Once the majority of sporangiospores had swollen, the germlings were separated from the medium by filtration through sterile gauze and then ground with liquid nitrogen for use in analytical sensitivity experiments.

### Human and mouse blood

This study was carried out in accordance with the recommendations of Management of Laboratory Animal, Laboratory Animal Welfare and Ethics Committee, Academy of Military Medical Sciences, China, and the protocol was approved by this committee (IACUC-13-2016-002). Mice blood was acquired from C57BL/6 mice, which were obtained from Animal Center of Academy of Military Medical Sciences. Human simulated blood (Product No. A7930) was purchased from Beijing Solarbio Science & Technology Co., Ltd.

### DNA extraction methods

The extraction of DNA from fungal cultures was conducted using the Biospin Fungus Genomic DNA Extraction Kit (Bioer Technology, Hangzhou, China), and simulated clinical samples were processed using the DNeasy® Blood & Tissue Kit (Qiagen, Madrid, Spain) according to the manufacturer’s instructions. Elution was performed using 50 ml of elution buffer. The quantification of DNA was carried out using the DeNovix Spectrophotometer (DeNovix Inc., America). The identification of ITS sequences was accomplished using the pairwise sequence alignment tool of BLAST from NCBI.

### Primer and probe design

The primers and probes utilized in this study were designed based on a consensus sequence obtained from the mitochondrial *rnl* (encoding large-subunit-ribosomal-RNA) gene of *R. oryzae*, *M. circinelloides* and *A. glauca* sequences (GenBank accession numbers AY863212.1, KR809877.1, and KU196782.1). The software MEGA6 (version 6.06) was employed for this purpose. The design of primers and probes for real-time RPA and qPCR was performed using Primer Premier 5 (version 5.0.0). To ensure specificity, these primers (listed in Table 3) were subjected to BLAST analysis to confirm no cross-reactivity with human and non-targeted fungi. All primers were synthesized by Tsingke Biotechnology (Beijing, China).

**Table 3:** Primers (5′ to 3′) for real-time RPA and real-time PCR.

Primer name	Primer sequence
Mucorales-Mt-RPA F	AGGACATGGTTGAAGGATAGGGTTTCCTTATCTGG
Mucorales-Mt-RPA R	CTTAGAGGCCGTTACTTTACTCATGGAGGTTGAGC
Mucorales-Mt-RPA P	CCTTATCTGGACATAACTGAGGAGAGAA[FAM-dT]G[THF] [BHQ-dT]GACATGAGTAACGTAA[3′-block]^a^
Mucorales-Mt-qPCR F	AACCGACACTGGTCTGCTG
Mucorales-Mt-qPCR R	TCTTCTATTCTGTGCCACGAC
Mucorales-Mt-qPCR P	[FAM-dT]TCCCGAAGTTACGGAGTCATTTTGC[BHQ1-dT]

^a^FAM-dT, thymidine nucleotide carrying fluorescein; BHQ-dT, thymidine nucleotide carrying Black Hole Quencher; THF, tetrahydrofuran spacer; 3′ to block elongation.

### RPA and real-time PCR assay methods

The RPA reaction was conducted in a 50 μL volume using the Nucleic Acid Amplification Kit (Qitian, Jiangsu, China). The reaction mixture consisted of 5 μL Buffer VI, 2.1 μL (10 μM) of each primer, 0.6 μL of the probe, and 2.5 μL of magnesium acetate. All reagents, except for the DNA template and magnesium acetate were combined in a master mix. The master mix was briefly vortexed for a few seconds and then distributed into individual reaction tubes containing a freeze-dried pellet. To each tube, 1 μL of genomic DNA or 5 μL of DNA extracted from simulated clinical samples was added. Subsequently, magnesium acetate was pipetted onto the tube lid and centrifuged into the bottom of the tube. Then, the tubes were immediately inserted into Light Cycler 96 instrument (Roche Diagnostics, Mannheim, Germany). The reaction was conducted at 40°C for 20 min.

A qPCR reaction with a volume of 25 μL was carried out in the Light Cycler 96, consisting of 12.5 μL 2 × FsatFire qPCR PreMix (TIANGEN, Beijing, China), 10 μM each primer, 10 μM probe, RNase-free water and either 2 μL genomic DNA or 5 μL DNA elution extracted obtained from simulated clinical samples. The cycling conditions encompassed an initial preincubation step at 95°C for 1 min, followed by an amplification program consisting of 40 cycles: denaturation at 95°C for 5 s and annealing/extension at 58°C for 15 s. In each experimental trial, both negative and positive controls were incorporated. The threshold for determining positivity was established as a Ct value of <38.

### Analytical sensitivity and specificity evaluation

The analytical sensitivity of real-time RPA and qPCR techniques was evaluated by employing 10-fold dilutions of DNAs from various fungal species, including *R. oryzae*, *M. racemosus*, *A. glauca*, *Rh. miehei*, and *C. bertholletiae*, ranging from 1 fg/μL to 1 ng/μL. Each assay was conducted a minimum of three times and the average CT value was calculated to ensure the reliability of the threshold cycle value outcomes.

The specificity and potential cross-reactivity were assessed by utilizing DNA samples extracted from 13 species within the Mucorales order, encompassing 5 genera, as well as 14 non-targeted strains, and samples of human and mouse blood. The strains of order Mucorales are *Rhizopus* spp. (*Rhizopus oryzae*, *Rhizopus microspores*), *Mucor* spp. (*Mucor mucedo*, *Mucor circinelloides*, *Mucor racemosus*, *Mucor pusillus*, *Mucor fragilis*), *Lichtheimia corymbifera*, *Absidia glauca*, *Rhizomucor* spp. (*Rhizomucor miehei*, *Rhizomucor pusillus*), *Cunninghamella bertholletiae*, *and Actinomucor elegans*. The non-Mucorales stains were *Aspergillus fumigatus*, *Aspergillus terreus*, *Trichoderma aureoviride*, *Penicillium chrysogenum*, *Candida albicans*, *Candida glabrata*, *Rhodotorula mucilaginosa*, *Cryptococcus neoformans*, *Alternaria alternata*, *Paraconiothyrium brasiliense*, *Fusarium oxysporum*, *Cladosporium cladosporioides*, *Acrophialophora levis*, *Escherichia coli*, and mammalian whole blood from mice and human.

In order to assess the potential impact of contamination from other strains on the sensitivity of detection, we introduced clinically significant pathogenic fungi of *A. fumigatus* or *C. albicans* (10 ng genomic DNA) into 10-fold dilutions of *R. oryzae*, *M. racemosus, A. glauca*, *Rh. miehei* and *C.bertholletiae* genomic DNA (ranging from 1 ng/μL to10 fg/μL).

### Preparation of simulated serum and blood samples

To simulate clinical patient samples, a 10-fold dilution series of 1 × 10^6^ to 1 × 10^1^ sporangiospores of *R. oryzae, M. racemosus, A. glauca, Rh. miehei,* and *C. bertholletiae* were introduced into 200 μL of simulated blood and serum. DNA from saline samples containing the aforementioned sporangiospores series served as the control group. All procedures were conducted within a class II biosafety cabinet to mitigate the risk of contamination arising from environmental fungal spores.

### Statistical analysis

Statistical analysis was conducted using GraphPad Prism 5 software (GraphPad Software, La Jolla, CA). The data in Table 2 and Supplementary Tables S3, S4 was represented as the mean ± standard deviation of the mean from at least three independent experiments, while the data in Figure 2 was represented as the mean.

## Data availability statement

The original contributions presented in the study are included in the article/Supplementary material, further inquiries can be directed to the corresponding authors.

## Ethics statement

Ethical approval was not required for the studies on humans in accordance with the local legislation and institutional requirements because only commercially available established cell lines were used. The animal study was approved by Laboratory Animal Welfare and Ethics Committee, Academy of Military Medical Sciences, China. The study was conducted in accordance with the local legislation and institutional requirements.

## Author contributions

RX: Investigation, Methodology, Project administration, Validation, Writing – review & editing, Formal analysis, Data curation, Software, Writing – original draft. DL: Formal analysis, Investigation, Methodology, Project administration, Validation, Writing – review & editing, Conceptualization, Resources, Supervision. JZ: Methodology, Project administration, Resources, Writing – review & editing. HZ: Data curation, Investigation, Software, Writing – review & editing. HC: Investigation, Methodology, Resources, Supervision, Writing – review & editing. YJ: Conceptualization, Data curation, Project administration, Software, Writing – review & editing. FC: Conceptualization, Data curation, Formal analysis, Methodology, Project administration, Resources, Supervision, Writing – review & editing. LH: Conceptualization, Funding acquisition, Investigation, Project administration, Resources, Supervision, Writing – review & editing.

## Abbreviations

RPA: recombinase polymerase amplification
qPCR: quantitative real-time polymerase chain reaction
LoD: limit of detection
CFU: colony forming unit
HSCT: hematopoietic stem cell transplant
COVID-19: corona virus disease 2019
CAM: COVID-19-associated mucormycosis
IFD: invasive fungal disease
PCR: polymerase chain reaction
HRM: high-resolution melting
ITS: internal transcribed spacer
FTR: high-affinity iron permease
cotH: spore coating encoding protein
BALF: bronchoalveolar lavage fluid
FFPE: formalin-fixed, paraffin-embedded tissue
RCA: rolling circle amplification
ESI-MS: electrospray-ionization mass spectrometry
LFS: lateral flow strips
PBS: phosphate-buffered saline
Ct value: threshold cycle value
