# Supplementary material for: Rapid detection of Mucorales based on recombinase polymerase amplification and real-time PCR
Source: Front Microbiol. 2023 Oct 20;14:1273073. doi: 10.3389/fmicb.2023.1273073 (PMC10635347; doi:10.3389/fmicb.2023.1273073)
Supplement: Supplementary file 1 [file Table_1.docx]

**SUPPLEMENT**

**Table S1** Molecular methods for detection of mucormycosis

| Method | Target | Product length (bp) | Sampels resource | Specific level to be identified | Sensitivity | Specifity | Cross reactivity | LoD | Time | Reference |
| --- | --- | --- | --- | --- | --- | --- | --- | --- | --- | --- |
| PCR | 18S rRNA | NA | Blood samples (n=4) | *Rhizopus spp.*, *Rhizomucor spp.*, *Mucor spp.*, *Lichtheimia spp.*, *Cunninghamella spp.* | 100% | NA | NA | NA | NA | [^25^](#_ENREF_25) |
|  | CotH | NA | Plasma, urine, BALF samples from mice | order Mucorales (including *R. delemar*, *R. oryzae*, *L. corymbifera*, *M. circinelloides*, *C. bertholletiae*) | 90% | 100% | NA | NA | NA | [^15^](#_ENREF_15) |
| Multiplex PCR | ITS | 448, 525,  663, 272 | FFPE (n=3),  serum (n=1) | *R. oryzae, R. azygosporus*/  *R. microsporus*, *R. stolonifer*, *R. schipperae* | 100% | NA | None | NA | NA | [^26^](#_ENREF_26) |
| Seminested PCR | 18S rRNA | 175-177 | FFPE (n=52) | *Rhizomucor spp.*, *M. hiemalis*,  *L. corymbifera*, *R. arrhizus* | 59% | 100% | NA | 0.1 fg  plasmid DNA | NA | [^27^](#_ENREF_27) |
|  | ITS1-5.8s-ITS2 | NA | FFPE (n=110) | *R. oryzae*, *L. corymbifra*,  *S. vasiformis*, *R. microsporus* | 88% | 89% | NA | NA | NA | [^28^](#_ENREF_28) |
| PCR +  Sequencing | ITS and/or D1/D2 regions | NA | Fungi cultures | *R. microsporus*, *Rh. pusillus*,  *C. bertholletiae*, *M. fragilis*, *S. racemosum, Lichtheimia spp.*  (*L. corymbifera*, *L. ramosa*) | 100% | NA | NA | NA | NA | [^29^](#_ENREF_29) |
| PCR + RFLP | 18S rRNA | NA | Clinical samples (n=2) | *Rhizopus spp*., *Rhizomucor spp.*, *Mucor spp.*, *Absidia corymbifera* | 100% | NA | None | NA | NA | [^30^](#_ENREF_30) |
|  | FTR1 | 585-740 | Fungi cultures | *Syncephalastrum spp.*, *Rhizomucor spp*., *R. oryzae, R. schipperae*, *R. microsporus var. rhizopodiformis*,  *R. microsporus var. oligosporus*, *R. niveus*, *R. stolonifer* | 100% | 100% | None | NA | NA | [^20^](#_ENREF_20) |
| PCR + ESI-MS | mtSSU rRNA, SSU rRNA, mt cytB, SSU rRNA | 90-136 | Fungi cultures | genus / species | 67% | NA | NA | NA | NA | [^31^](#_ENREF_31) |
|  | cytochrome *b* gene and 18S rRNA gene | NA | biopsy specimens (n=19) | *A. elegans*, *L. corymbifera*,  *R. arrhizus*, *L. ramosa*,  *M. circinelloides*, *R. microsporus* | NA | NA | NA | NA | 6h | [^32^](#_ENREF_32) |
| PCR +  Oligonucleo-  tide array | ITS 1  and ITS 2 | NA | Fungi cultures (n=397) | *L. corymbifera*, *C. bertholletiae*, *Cunninghamella spp.*, *R. oryzae*,  *Rh. pusillus* | 98.30% | 98.10%[^31^](#_ENREF_31) | NA | NA | 24h | [^33^](#_ENREF_33) |
| qPCR | 18S rRNA | 105, 118,  105 | Serum (n=51) | *Rhizomucor spp.*, *Lichtheimia spp.*, *Mucor/Rhizopus spp.* | 90% | NA | None | 3.7-15 fg | 2h | [^19^](#_ENREF_19) |
|  | 18S rRNA | 162 | Serum (n=8), BALF（n=2） | *Cunninghamella spp.* | 80% | NA | NA | NA | NA | [^34^](#_ENREF_34) |
|  | 18S rRNA | 175 | Tissue (n=12)/ Serum (n=268） | order Mucorales (including *Rhizopus spp.*, *Lichtheimia spp.*, *Rhizomucor spp.*, *Mucor spp.*, *Actinomucor spp.*) | 91% / NA | 100% / NA | NA | NA | NA | [^22^](#_ENREF_22) |
|  | 18S rRNA | NA | serum,blood,  cerebrospinal fluids, roncho-pulmonary, biopsy speci-  mens (n=364) | order Mucorales (including 8 different genera and 11 different species） | 73.33% -  100% | 98.1% - 100% | None | 3 DNA copy | NA | [^35^](#_ENREF_35) |
|  | 18S rRNA 28S rRNA | 175 107 | FFPE (n=3),  fresh specimens (n=14) | order Mucorales (including 8 genera, 12 species, sequencing of amplicons of the 18S assay allowed identification to genus level) | 90% | 100% | None | 3 - 64 fg | NA | [^21^](#_ENREF_21) |
| qPCR+HRM | 28S rRNA | 180, 148 | BALF, lung tissue from  rabbits | qPCR-1：*R. microsporus*, *Rh. pusillus*, *M. circinelloides* qPCR-2：*C. bertholletiae* | 99-100% 100% | NA | None | 10 /  10-100 copies | 2h | [^24^](#_ENREF_24) |
|  | cytochrome b | 167 | Cuture isolates/ Fresh tissue/ FFPE | order Mucorales (including *lichthemia spp.*, *Apophysomyces spp.*, *Cunninghamella spp.*, *Mucor spp.*, *Rhizopus spp.*, *Saksenaea spp.*), *Cunninghamella spp.*, *Rhizopus spp.*, *Saksenaea spp.* | 100% / 100% /  56% | 92% / 100% / 100% | None | 10  targrts/μL | NA | [^23^](#_ENREF_23) |
|  | 18S rRNA | NA | BALF (n=99) | *Rhizopus spp.*, *Rh. pusillus*,  *L. corymbifera*, *Mucor spp.* | 93% | 100% | NA | 1fg / μL | 5h | [^36^](#_ENREF_36) |
|  | mitochondrial *rnl* | 124 | FFPE (n=21) | *R. microsporus*, *M. circinelloides,* *R. arrhizus*, *Lichtheimia spp.* ,Complex (*L. corymbifera*, *L. ramosa*) | 71.40% | NA | None | 50 fg/μL | NA | [^37^](#_ENREF_37) |
| Multiplex real-time PCR | ITS1 or ITS2 | 192, 187, 263 | Biopsy (n=12) | *R. oryzae*, *R. microsporus*,  *Mucor spp.* | 100% | 100% | None | 1 fg / μL | 2–3 h | [^17^](#_ENREF_17) |
|  | ITS | NA | FFPE (n=102) | *R. microsporus*, *R. oryzae*, *Mucor spp.*, *C. bertholletiae*, *Lichtheimia spp.*, *Syncephalastrum spp.*, *Rhizomucor spp*. | 62% | NA | None | NA | NA | [^38^](#_ENREF_38) |

**Table S2 Application of RPA assay in fungi**

| **RPA in detection** | **Target gene** | **Template** | **LoD** | **Specificity** | **Assay time** | **Detection method** | **Reference** |
| --- | --- | --- | --- | --- | --- | --- | --- |
| *Candida albicans* | ITS2 | DNA | 1 CFU per reaction  or 10^2^ fg /50 µl | 100% | 25 min | LFS-RPA | [^42^](#_ENREF_42) |
| *Candida tropicalis* | ITS2 | DNA | 9.94 CFU/µL | 100% | 20 min | LFS-RPA | [^43^](#_ENREF_43) |
| *Candida glabrata* | ITS2 | DNA | 10 CFU/50 µL | 100% | 20 min | LFS-RPA | [^44^](#_ENREF_44) |
| *Cryptococcus neoformans* | ITS | DNA | 0.64 pg per reaction | 95.80% | 10 min | LFS-RPA | [^45^](#_ENREF_45) |
| *Aspergillus fumigatus* | ITS | DNA | 3 copies | 91.70% | 30 min | CRISPR/Cas13a-RPA | [^46^](#_ENREF_46) |
| *Fusarium graminearum* | gaoA | DNA | 20 fg | - | 35 min | RPA-LFD | [^47^](#_ENREF_47) |
| *Microsporum canis,*  *Trichophyton mentagrophytes* | ITS | DNA | - | 100% | 30 min | CRISPR-Cas12a-RPA | [^48^](#_ENREF_48) |
| *Leptosphaeria maculans* | ITS | DNA | 4.7 copies/μL | - | 45 min | CRISPR-Cas12a-RPA | [^49^](#_ENREF_49) |

ITS: Internal transcribed spacer, gaoA: galactose oxidase gene, RPA: Recombinase polymerase amplification, LFS: Lateral flow strips, CRISPR/Cas13a: Clustered regularly interspaced short palindromic repeat-associated protein 13a, LFD: lateral flow dipstick, CRISPR-Cas12a: Clustered regularly interspaced short palindromic repeat-associated protein 12a, min: minute, -: Not mentioned.

**Table S3A** LoDs (ng) of Mucorales in spiked *A.fumigatus* or *C.albicans* specimens by real-time RPA

| Species | non-spiked | |  | *A.fumigatu*s-spiked | |  | *C.albicans*-spiked | |
| --- | --- | --- | --- | --- | --- | --- | --- | --- |
|  | LoD | Ct Value |  | LoD | Ct Value |  | LoD | Ct Value |
| *R. oryzae* | 10^-3^ | 8.39±0.39 |  | 10^-3^ | 6.4±1.07 |  | 10^-3^ | 8.97±2.77 |
| *M. racemosus* | 10^-3^ | 10.32±1.07 |  | 10^-3^ | 9.27±2.42 |  | 10^-3^ | 8.51±0.59 |
| *A. glauca* | 10^-3^ | 10.33±1.65 |  | 10^-3^ | 7.35±1.50 |  | 10^-3^ | 7.90±0.47 |
| *Rh. miehei* | 10^0^ | 6.9±1.01 |  | 10^0^ | 9.32±0.96 |  | 10^0^ | 9.19±0.06 |
| *C. bertholletiae* | 10^-3^ | 9.86±1.80 |  | 10^-3^ | 9.82±3.22 |  | 10^-3^ | 9.17±2.45 |

**Table S3B** LoDs (ng) of Mucorales in spiked *A.fumigatus* or *C.albicans* specimens by real-time PCR

| Species | non-spiked | |  | *A.fumigatu*s-spiked | |  | *C.albicans*-spiked | |
| --- | --- | --- | --- | --- | --- | --- | --- | --- |
|  | LoD | Ct Value |  | LoD | Ct Value |  | LoD | Ct Value |
| *R. oryzae* | 10^-4^ | 33.05±2.24 |  | 10^-4^ | 35.57±2.14 |  | 10^-4^ | 37.53±0.49 |
| *M. racemosus* | 10^-5^ | 36.26±1.53 |  | 10^-5^ | 36.62±1.58 |  | 10^-5^ | 36.53±0.30 |
| *A. glauca* | 10^-5^ | 35.84±0.67 |  | 10^-5^ | 34.75±2.38 |  | 10^-5^ | 35.76±1.41 |
| *Rh. miehei* | 10^-3^ | 35.46±1.40 |  | 10^-3^ | 35.53±1.10 |  | 10^-3^ | 36.85±1.43 |
| *C. bertholletiae* | 10^-4^ | 35.61±2.43 |  | 10^-4^ | 37.00±0.67 |  | 10^-4^ | 36.80±0.52 |

Each template contains 1 μL targeted species and 1 μL *A.fumigatus* or *C.albicans* (DNA concentration is 10 ng/μL) per reaction, and non-spiked template only contains 1 μL targeted species.

**Table S4A** Detection (CFU) of simulated clinical samples by real-time RPA^①^

| Specimen | Species | 10^6^ | 10^5^ | 10^4^ | 10^3^ | 10^2^ | 10^1^ |
| --- | --- | --- | --- | --- | --- | --- | --- |
| Saline | *R. oryzae* | 6.48±1.70 | 8.88±1.87 | 13.75±2.18 | 15.37±2.14 | ^②^ND | ND |
|  | *M. racemosus* | 4.44±1.40 | 6.12±0.90 | 10.08±2.98 | 13.27±3.86 | ND | ND |
|  | *A. glauca* | 4.40±0.97 | 5.84±0.58 | 10.26±2.74 | 15.69±2.62 | ND | ND |
|  | *Rh. miehei* | ^③^7.24 | ND | ND | ND | ND | ND |
|  | *C. bertholletiae* | 4.74±0.36 | 7.39±2.15 | 11.76±0.79 | ND | ND | ND |
| Serum | *R. oryzae* | 9.76±1.25 | 13.45±1.16 | ND | ND | ND | ND |
|  | *M. racemosus* | 7.23±1.74 | 9.93±4.81 | 12.04±4.33 | 14.79±3.93 | ND | ND |
|  | *A. glauca* | 8.54±2.82 | 10.06±2.75 | 12.98±1.41 | ^③^17.2 | ND | ND |
|  | *Rh. miehei* | ^③^12.87 | ND | ND | ND | ND | ND |
|  | *C. bertholletiae* | 7.35±1.05 | 10.41±0.13 | ^③^12.98 | ND | ND | ND |
| Blood | *R. oryzae* | 9.25±5.11 | 17.19±3.87 | ND | ND | ND | ND |
|  | *M. racemosus* | 9.23±1.78 | 11.04±1.65 | 13.89±4.06 | ND | ND | ND |
|  | *A. glauca* | 12.34±1.63 | 13.20±1.51 | ND | ND | ND | ND |
|  | *Rh. miehei* | 11.26±5.69 | ND | ND | ND | ND | ND |
|  | *C. bertholletiae* | 8.58±3.51 | 12.71±4.72 | ND | ND | ND | ND |

**Table S4B** Detection (CFU) of simulated clinical samples by real-time PCR^1^

| Specimen | Species | 10^6^ | 10^5^ | 10^4^ | 10^3^ | 10^2^ | 10^1^ |
| --- | --- | --- | --- | --- | --- | --- | --- |
| Saline | *R. oryzae* | 26.15±0.51 | 30.34±0.39 | 34.50±0.59 | 35.04±2.88 | 35.57±1.24 | 35.91±0.54 |
|  | *M. racemosus* | 19.87±0.84 | 21.70±1.12 | 27.19±0.33 | 30.23±0.27 | 34.83±0.47 | 35.35±0.63 |
|  | *A. glauca* | 22.27±0.85 | 25.78±1.11 | 30.96±0.62 | 33.75±0.90 | 34.41±0.64 | 35.84±0.79 |
|  | *Rh. miehei* | 28.28±1.04 | 33.16±1.00 | 35.62±0.21 | 36.64±0.64 | ND | ND |
|  | *C. bertholletiae* | 24.28±2.52 | 26.01±0.95 | 30.83±1.80 | 35.65±0.48 | 35.92±1.24 | 36.14±0.52 |
| Serum | *R. oryzae* | 27.68±2.59 | 30.18±1.96 | 33.25±2.53 | 34.65±0.90 | 34.71±0.50 | 35.66±1.77 |
|  | *M. racemosus* | 23.56±1.56 | 25.82±1.39 | 27.94±0.67 | 30.17±0.30 | 30.89±1.02 | 32.07±0.82 |
|  | *A. glauca* | 25.49±2.46 | 27.88±2.71 | 30.54±2.23 | 33.88±0.97 | 34.38±0.78 | 34.64±0.75 |
|  | *Rh. miehei* | 30.16±0.36 | 31.93±1.39 | 32.12±1.82 | 33.27±2.24 | ND | ND |
|  | *C. bertholletiae* | 25.06±1.27 | 27.72±1.79 | 31.59±2.64 | 32.10±2.54 | 33.58±1.50 | 36.95±0.74 |
| Blood | *R. oryzae* | 27.67±1.52 | 29.45±0.13 | 30.20±0.90 | 31.98±1.28 | 32.86±1.09 | ND |
|  | *M. racemosus* | 23.91±1.89 | 26.24±2.40 | 29.12±2.90 | 31.33±2.24 | 33.18±0.68 | ND |
|  | *A. glauca* | 25.38±2.29 | 27.96±2.22 | 31.77±3.16 | 33.50±0.70 | 34.22±1.15 | ND |
|  | *Rh. miehei* | 29.17±2.43 | 31.90±3.54 | 31.87±3.99 | 32.23±3.36 | ND | ND |
|  | *C. bertholletiae* | 24.76±1.34 | 28.34±1.45 | 31.26±0.79 | 31.84±1.45 | 32.71±2.31 | ND |

① Aliquots of 200 µL samples were inoculated with a ten-fold dilution series of 1 × 10^6^ - 1 × 10^1^ CFU conidia of *R. oryzae*, *M. racemosus*, *A. glauca*, *Rh. miehei* and *C. bertholletiae.* Each assay was repeated in 3 different batch of samples.

② ND, not detected.

③ There was only one positive result in three assays.
